# Impact of uranium uptake on isotopic fractionation and endogenous element homeostasis in human neuron-like cells

**DOI:** 10.1038/s41598-018-35413-4

**Published:** 2018-11-21

**Authors:** Eduardo Paredes, Emilie Avazeri, Véronique Malard, Claude Vidaud, Pascal E. Reiller, Richard Ortega, Anthony Nonell, Hélène Isnard, Frédéric Chartier, Carole Bresson

**Affiliations:** 1 Den – Service d’Etudes Analytiques et de Réactivité des Surfaces (SEARS), CEA, Université Paris-Saclay, F-91191 Gif sur Yvette, France; 2 CEA, DRF, Biosciences and biotechnologies institute (BIAM), F-30200 Bagnols-sur-Cèze, France; 3 Laboratory of Protein-Metal Interactions (LIPM), Institute of Biosciences and Biotechnologies of Aix Marseille (BIAM), UMR7265 CEA – CNRS - Aix Marseille Univ, CEA Cadarache, F-13108 Cadarache, France; 4 University of Bordeaux, CENBG, UMR 5797, F-33170 Gradignan, France; 5 CNRS, IN2P3, CENBG, UMR 5797, F-33170 Gradignan, France; 6 Den – Département de Physico-Chimie (DPC), CEA, Université Paris-Saclay, F-91191 Gif sur Yvette, France

## Abstract

The impact of natural uranium (U) on differentiated human neuron-like cells exposed to 1, 10, 125, and 250 µM of U for seven days was assessed. In particular, the effect of the U uptake on the homeostatic modulation of several endogenous elements (Mg, P, Mn, Fe, Zn, and Cu), the U isotopic fractionation upon its incorporation by the cells and the evolution of the intracellular Cu and Zn isotopic signatures were studied. The intracellular accumulation of U was accompanied by a preferential uptake of ^235^U for cells exposed to 1 and 10 µM of U, whereas no significant isotopic fractionation was observed between the extra- and the intracellular media for higher exposure U concentrations. The U uptake was also found to modulate the homeostasis of Cu, Fe, and Mn for cells exposed to 125 and 250 µM of U, but the intracellular Cu isotopic signature was not modified. The intracellular Zn isotopic signature was not modified either. The activation of the non-specific U uptake pathway might be related to this homeostatic modulation. All together, these results show that isotopic and quantitative analyses of toxic and endogenous elements are powerful tools to help deciphering the toxicity mechanisms of heavy metals.

## Introduction

Identifying the metabolic pathways of toxic heavy metals and the subsequent altered metabolic processes following an exposure is of prime concern for the understanding of the mechanisms involved in their toxic effects. Such knowledge is critical for the improvement of diagnostic strategies, as well as for the development of more efficient curative and detoxification treatments. The study of the impairment of the homeostasis of endogenous elements following an exposure as well as the isotopic analysis of the toxic and endogenous elements are tools that can provide invaluable clues to be able to face such a challenging topic. Indeed, the study of metal isotopic fractionations in biological fluids and tissues has recently gained a great interest for toxicological investigations^1^, as well as in metabolic^2–4^ studies and biomedical^5–7^ applications. For instance, cancer^8–10^ and neurodegenerative diseases^11,12^ have been found to induce variations in the Cu and Zn isotope ratios in fluids and tissues, opening new perspectives for the use of metal isotopic signatures as biomarkers of diseases^8,11,13,14^, for early diagnosis^9^, and for the follow-up of patients^15^. Since isotopic fractionations can occur during different metabolic processes^7^, significant isotopic variations can result from the alteration of the processes involving these endogenous elements.

Attempts have been made to identify the altered metabolic processes connected with the *in vivo* isotopic fractionations in biological fluids and tissues of patients^9^. However, this task is cumbersome due to the possible additional isotopic variations among individuals associated to their age^16^ or their dietary habits^17,18^. Animal^1,19^ and *in vitro* cultured human cell models^20–23^ seem to be more promising tools for isotopic variations studies, since the effect of these variables is much smaller or negligible. Nevertheless, there are only a few investigations on isotopic fractionations in *in vitro* cultured human cell lines. Bondanese *et al*.^21^ showed that Cu was isotopically heavier in a cancerous liver cell model under hypoxic conditions compared to normoxic conditions. This was in agreement with the enrichment in the lighter ^63^Cu isotope found in the blood of cancer patients^8,9^, suggesting that *in vivo* tumor hypoxic conditions lead to the enrichment in the heavier ^65^Cu isotope in the cancerous tissue. A more recent publication showed that the exposure of the same cell model to oxidative stress conditions also led to heavier intracellular Cu isotopic signatures^22^. The same authors also demonstrated the preferential incorporation of the lighter Fe isotopes in an intestinal cell model^23^, in line with previous *in vivo* observations^4^. Finally, in our recent study aimed at identifying potential U uptake pathways in a human cell model differentiated into neuron-like cells exposed to 10 µM of natural U for 7 days^20^, we measured an intracellular enrichment of the lighter ^235^U isotope by 0.38 ± 0.13‰. These isotopic data allowed us to suggest two potential U uptake processes in agreement with the direction of the U isotopic fractionation^20^: (i) an equilibrium process consisting in the U uptake through the coordination of uranyl (UO_2_^2+^) to a high-affinity uranium transport protein; and (ii) the kinetically-controlled facilitated transmembrane diffusion of U species.

These first results prompted us to investigate further the isotopic fractionation of U resulting from its uptake by neuron-like cells exposed to variable natural U concentrations of 1, 10, 125, and 250 µM for 7 days. Since intracellular U could impair the homeostasis of endogenous elements, we evaluated the influence of U uptake on the homeostatic modulation of some endogenous elements (Mg, P, Mn, Fe, Zn, Cu), and studied the evolution of the intracellular Cu and Zn isotopic signatures in cells exposed to different natural U concentrations. According to the literature related to high-precision isotopic analysis of essential elements in biological media^5^, Ca, Cu, Fe and Zn are the most relevant elements for such a study. Since Ca and Fe were not present at high enough amounts in the samples, we focused only on the determination of the intracellular isotopic signatures of Cu and Zn, following uranium exposure. The results obtained allowed us to propose potential U uptake pathways as a function of the exposure U concentration and to discuss the effect of U uptake on the homeostasis of endogenous elements.

## Results

### Intracellular U accumulation and effect on the homeostasis of endogenous elements

**Figure 1:**
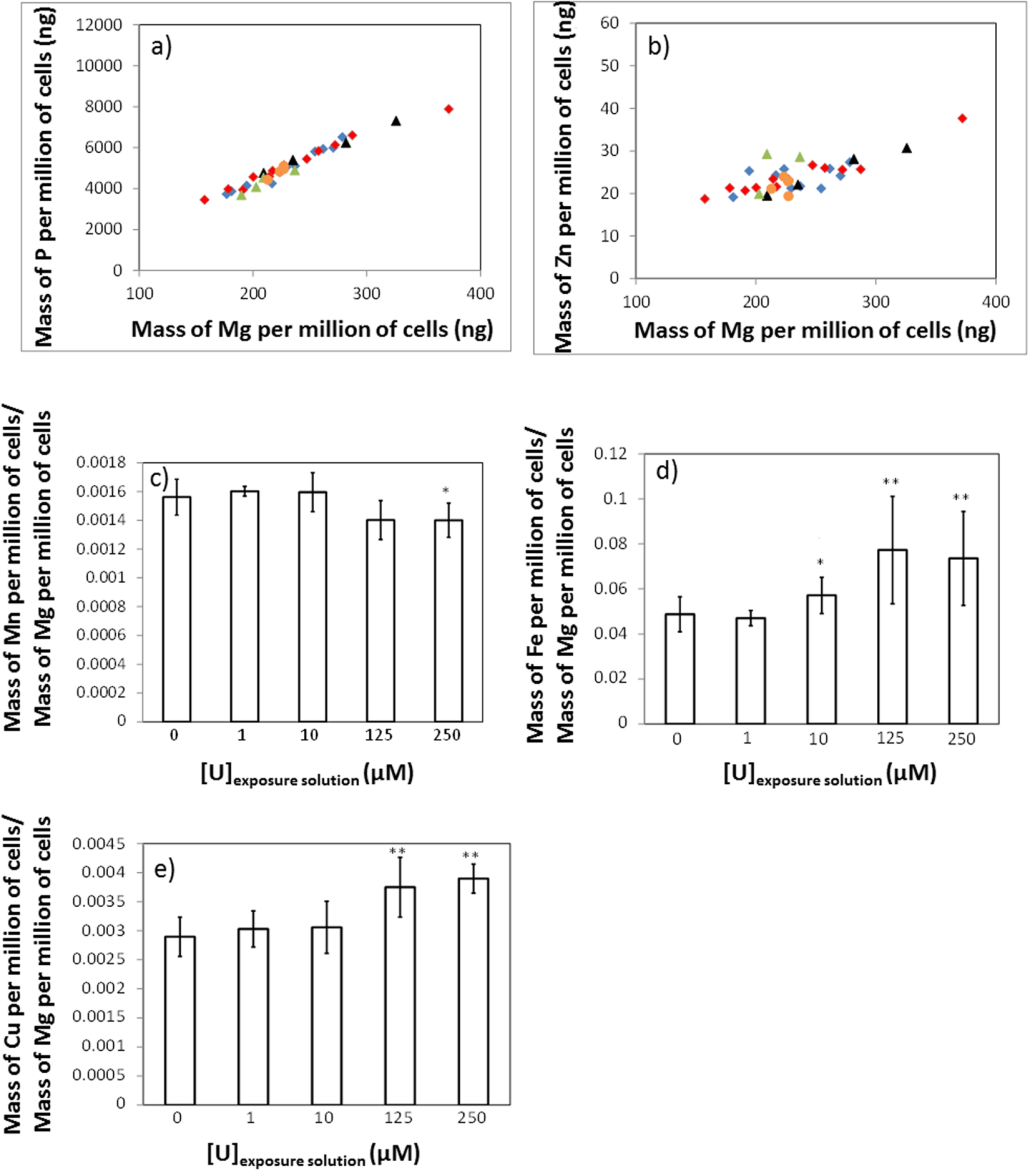
Correlation between the mass of element per million of cells for P and Mg (**a**) and for Zn and Mg (**b**) for individual cell samples exposed to U concentrations of 0 µM (blue diamonds), 1 µM (black triangles), 10 µM (red diamonds), 125 µM (green triangles) and 250 µM (orange circles). (**c**–**e**) show the average values of the mass of element per million of cells relative to the mass of Mg per million of cells in cell samples exposed to U concentrations of 0, 1, 10, 125, and 250 µM for Mn (**c**), Fe (**d**), and Cu (**e**). Error bars correspond to the standard deviation of 12, 4, 14, 5 and 5 replicates for cell samples exposed to 0, 1, 10, 125, and 250 µM of U, respectively. The results showing a significant difference with regard to cell samples exposed to 0 µM of U, according to a non-parametric Mann-Whitney test, are shown: *(P < 0.05); **(P < 0.01).

The intracellular amounts of U and endogenous elements (Mg, P, Mn, Fe, Zn, and Cu) in SHY-5Y neuron-like cells exposed to 0, 1, 10, 125, and 250 µM of natural U for seven days are shown in Table 1. These exposure conditions corresponded to sub-cytotoxic or moderately toxic U effects, with 15% inhibition of cell metabolism for 250 µM of natural U^24^. The results are expressed as total mass of element per million of cells. This table reveals that the cells incorporated approximately 0.2 and 0.7% of the total mass of U contained in the exposure solutions, for cells exposed to 1 and 10 µM of natural U, respectively. For cells exposed to 125 and 250 µM of natural U, this percentage increased up to *approx*. 2%. The intracellular amounts of Fe, Cu, and Zn, the most studied endogenous elements in mammalian cell lines, were of the same order of magnitude than those found in the literature for other cell lines^25^. The uncertainties of the results were quite large, with relative standard deviations (RSD) for different replicates of cells exposed to the same U concentration of 21% on average. Three main sources of uncertainty were identified when expressing the results in ng of element per million of cells: (i) the variability of the elemental content in cell samples for different replicates; (ii) the uncertainty associated to the analytical procedure; and (iii) the uncertainty of cell counting. The RSDs of the number of cells with the cell counting method used in this work were typically 10–20%, which were similar to the RSDs of the results obtained for cell samples exposed to the same U concentration, as it can be calculated from the data presented in Table 1. For instance, for Mg and P, the two most abundant intracellular elements quantified in this work, the RSDs ranged from 3 to 21% and from 6 to 23%, respectively. The good correlation between the values for P and Mg, and for Zn and Mg, respectively, in different replicates (Fig. 1a,b) confirms that the uncertainty of cell counting was the main source of this spread. Furthermore, all the results fall on the same straight line, meaning that the intracellular concentrations of these three elements did not change with the U concentration in the exposure solution. In order to eliminate the contribution of cell counting to the uncertainties, the results were expressed relative to the mass of Mg per million of cells (Fig. 1c–e). Mg was selected to normalize the results for two reasons. On the one hand, it was one of the most abundant elements analyzed, together with P, and its concentration was determined with a better precision than for the other elements. On the other hand, unlike P, which has low ionization efficiency in the plasma because of its high ionization potential, Mg has an ionization potential much closer to the other elements. According to a non-parametric Mann-Whitney test, a significant difference with regard to control cells was found for Cu in samples exposed to 125 and 250 µM of natural U (Fig. 1e); for Mn in samples exposed to 250 µM of natural U (Fig. 1c); and for Fe in samples exposed to 10, 125, and 250 µM of natural U (Fig. 1d). The intracellular concentrations of the other endogenous elements (Mg, P, and Zn) remained unchanged upon U exposure (data not shown).

**Table 1:** Content of Mg, P, Mn, Fe, Zn, Cu, and U expressed in ng per million of cells (±1 SD), in differentiated human neuron-like cells exposed to: 0 µM (control cells), 1, 10, 125, and 250 µM of natural uranium.

		U exposure conditions: extracellular concentration ( µM)				
		0	1	10	125	250
Intracellularcontent(ng/10^6^ cells)	***Mg***	230 ± 30	260 ± 50	240 ± 50	210 ± 20	223 ± 6
	***P***	4900 ± 900	5900 ± 1100	5100 ± 1200	4300 ± 500	4900 ± 300
	***Mn***	0.36 ± 0.06	0.42 ± 0.08	0.37 ± 0.08	0.31 ± 0.04	0.31 ± 0.03
	***Fe***	11 ± 2	12 ± 2	13 ± 2	18 ± 7	16 ± 5
	***Zn***	23 ± 3	25 ± 5	24 ± 5	26 ± 5	22 ± 2
	***Cu***	0.67 ± 0.12	0.8 ± 0.2	0.7 ± 0.2	0.8 ± 0.2	0.87 ± 0.06
	***U***	**ND**	**0.46** ± **0.10**	**20** ± **12**	**800** ± **140**	**1790** ± **70**

^#1^The number of replicates was 12 for control cells and 4, 14, 5 and 5 for cells exposed to 1, 10, 125, and 250 µM of uranium, respectively. ND = Not detected.

### Isotopic fractionation of uranium during the uptake process

**Figure 2:**
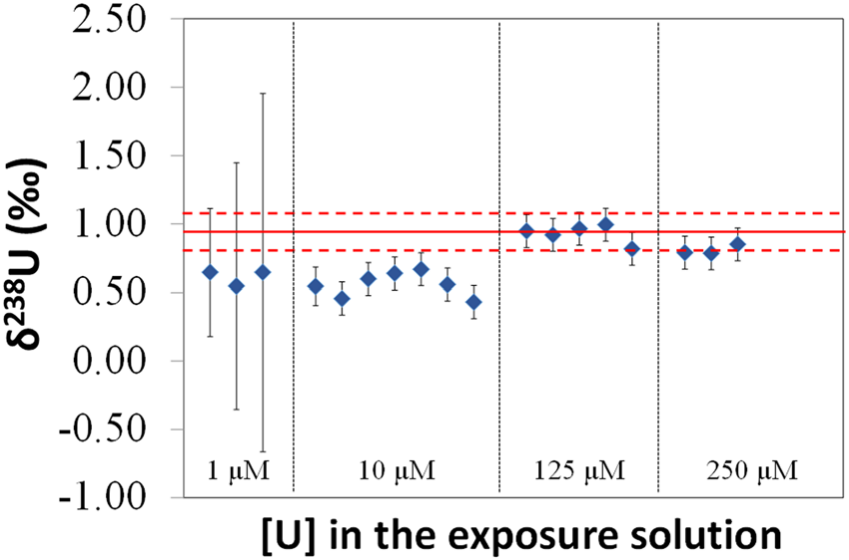
δ^238^U (‰) values for intracellular U in cell samples exposed to natural U concentrations of 1, 10, 125, and 250 µM with regard to the average of the n(^238^U)/n(^235^U) determined for the IRMM–184 certified reference material in the bracketing solution analyzed just before and after the sample. Error bars correspond to the expanded uncertainties (k = 2). The 7 values for 10 µM U exposure concentration correspond to those presented in reference 20. Plain and dashed red lines correspond to the average and the reproducibility (2 SD) of δ^238^U (‰) values determined for extracellular U in 14 independent exposure solutions containing U concentrations from 1 to 250 µM.

Figure 2 shows the δ^238^U (‰) values (see Equation 1) for intracellular U in cell samples exposed to 1, 10, 125 and 250 µM of natural U. For comparison, the δ^238^U (‰) of the extracellular U in the exposure solutions, δ^238^U_extra_, which was 0.92 ± 0.14‰ (2 SD, n = 16) is given (red line). This value was not significantly different compared to the δ^238^U (‰) in the initial U stock solution used to prepare the exposure solutions, which was 0.89 ± 0.12‰ (2 SD, n = 7). An isotopic fractionation of 0.36‰ between the extracellular, δ^238^U_extra_, and the intracellular U was previously observed for cell samples exposed to 10 µM of natural U^20^ (δ^238^U_intra,10µM_ = 0.56 ± 0.13‰, 2 SD, n = 7), with a preferential intracellular incorporation of the ^235^U isotope. A similar shift was determined in this work for cell samples exposed to 1 µM of natural U (δ^238^U_intra,1µM_ = 0.61 ± 0.12‰, 2 SD, n = 3), but the associated expanded uncertainties (U_c_, k = 2) for individual results were much higher because of the small intracellular U amounts^26^, ranging from 10 to 15 ng. Figure 2 also shows that the U isotopic fractionation was no longer observed for cell samples exposed to higher U concentrations, 125 and 250 µM, with δ^238^U_intra,125µM_ of 0.93 ± 0.14‰ (2 SD, n = 5) and δ^238^U_intra,250µM_ of 0.81 ± 0.08‰ (2 SD, n = 3), respectively.

### Intracellular Cu and Zn isotopic signatures following exposure of the cells to different uranium concentrations

**Figure 3:**
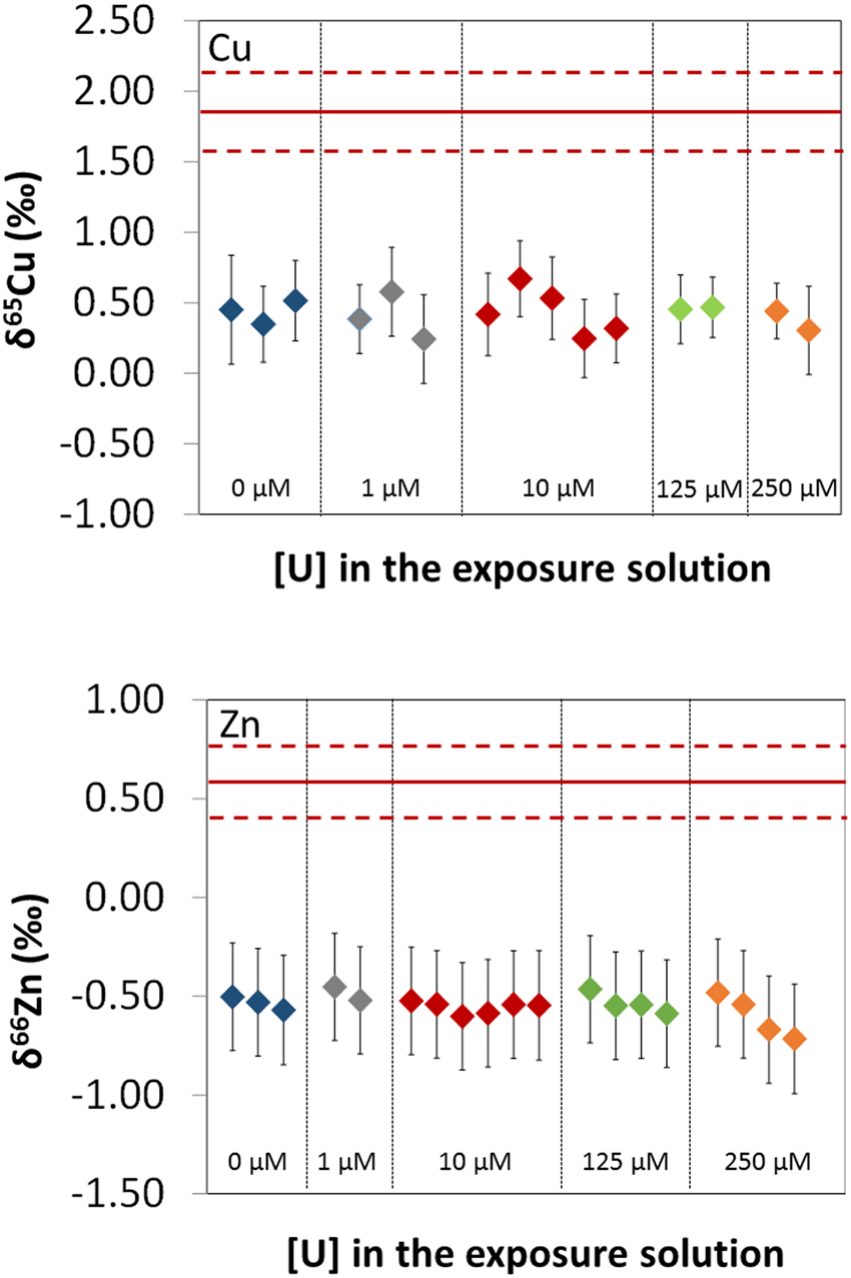
δ^65^Cu (‰) values for intracellular Cu and δ^66^Zn (‰) values for intracellular Zn in cell samples exposed to U concentrations of 0, 1, 10, 125, and 250 µM. The δ^65^Cu (‰) and δ^66^Zn (‰) values are expressed relative to the average n(^65^Cu)/n(^63^Cu) determined for the ERM^®^–AE633 and the average n(^66^Zn)/n(^64^Zn) determined for the IRMM–3702 certified reference materials, respectively, in the bracketing solution analyzed just before and after the sample. Error bars correspond to the expanded uncertainties (k = 2). Plain and dashed red lines correspond to the average and the reproducibility (2 SD) of δ^65^Cu (‰) and δ^66^Zn (‰) values in the exposure solutions (n = 9 for δ^65^Cu and n = 17 for δ^66^Zn).

Figure 3 shows the δ^65^Cu (‰) and δ^66^Zn (‰) values for intracellular Cu and Zn in cell samples exposed to U concentrations of 0, 1, 10, 125 and 250 µM. It is clearly observable that the U uptake by the cells did not induce any significant change within uncertainties in the isotopic signatures of intracellular Cu and Zn, whatever the U concentration in the exposure solution.

The average intracellular Cu isotopic signature, δ^65^Cu_intra_, of the population of results obtained from the control cells and after cell exposure to the four U concentrations, was 0.43 ± 0.28‰ (2 SD, n = 15). Since the extracellular Cu isotopic signature, δ^65^Cu_extra_, was 1.85 ± 0.28‰ (2 SD, n = 9), this result indicates that the lighter ^63^Cu isotope was preferentially incorporated in the differentiated neuron-like cells, with an isotopic fractionation of 1.42‰. This result is similar to the isotopic fractionation of approximately 1.5‰ recently determined between the extracellular and the intracellular media for a liver cell model^22^. In the case of Zn, the intracellular Zn isotopic signature, δ^66^Zn_intra_, was −0.55 ± 0.13‰ (2 SD, n = 19), whereas the extracellular Zn isotopic signature, δ^66^Zn_extra_ was 0.59 ± 0.18‰ (2 SD, n = 19). Therefore, the lighter ^64^Zn isotope was also preferentially incorporated, with an isotopic fractionation of 1.14‰. Several processes taking place during the Cu^27,28^ and Zn^29,30^ uptake by the cells may lead to the preferential incorporation of the light Cu and Zn isotopes, including the exchange reactions of the Cu and Zn from their species in the exposure solution to the corresponding protein transporters, the exchange reactions of the Cu and Zn between the amino-acids of the different binding domains of the protein transporters, and, in the case of Cu, the reduction of Cu(II) to Cu(I) previous to the Cu uptake. These processes will be discussed in a future publication investigating the isotopic fractionation of these elements in different human cell models.

## Discussion

U is a ubiquitous element in the Earth’s crust, and humans are potentially exposed to small U amounts. The brain is a potential target of U^31^ and associated neurotoxic effects have been suspected in humans^32^. However, there is very little knowledge on the metabolic processes involving U at cellular and molecular levels, and even less on cellular U uptake processes. It must be pointed out that the U incorporation in its natural or depleted forms, by different types of cultured mammalian cells, including human cell lines, has been previously observed by imaging techniques^33–35^. These studies were performed at U exposure concentrations of typically hundreds of µM and always higher than 10 µM, with times of exposure lower than 48 h. In the present work, we have studied for the first time the impact of natural U on differentiated human neuron-like cells, after exposure to U concentrations as low as 1 µM for seven days. These exposure duration and concentrations were selected in order to evaluate the impact of small U amounts over a long exposure period. The U accumulation by these cells at any of the considered concentrations is demonstrated in Table 1, with an increase in the percentage of extracellular U incorporated by the cells as the extracellular U concentration increased.

A concentration-dependent U isotopic fractionation between the extracellular and the intracellular media was emphasized at low U concentrations. The differentiated neuron-like cells were enriched in ^235^U with regard to the exposure solutions at 1 and 10 µM of U, whereas there was not any significant isotopic fractionation in the case of cells exposed to 125 and 250 µM of U. Three potential hypotheses to explain this concentration-dependent isotopic fractionation could be suggested: (i) a change in the U speciation in the exposure solution when increasing the extracellular U concentration; (ii) the activation of a U efflux process triggered by a threshold intracellular U concentration; and (iii) the activation of a second U incorporation pathway above a threshold extracellular U concentration in the exposure solution.

**Figure 4:**
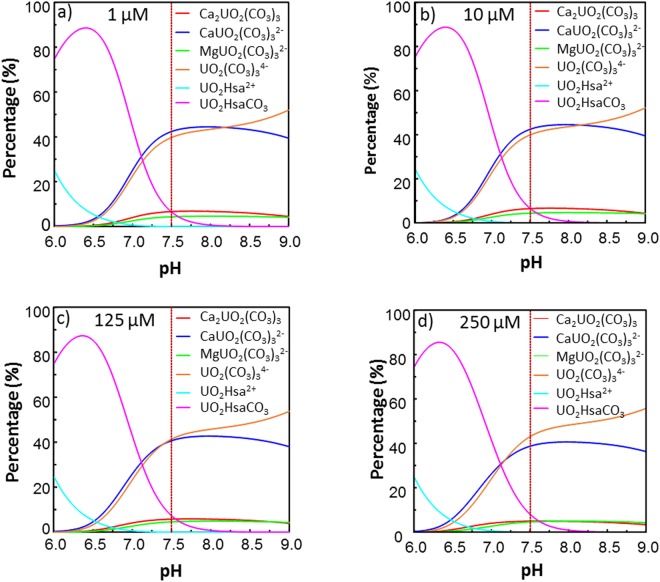
Theoretical speciation diagrams of U at 1 (**a**), 10 (**b**), 125 (**c**), and 250 (**d**) µM in the exposure solutions. Hsa stands for human serum albumin. The pointed lines show the percentage of the U species at the pH of the exposure solutions (7.5). Species representing less than 5% in total are not plotted.

The effect of the speciation of U in the exposure solution on the U cytotoxicity, as well as on the U amounts incorporated by cultured mammalian cells, has been evidenced in previous studies^35–38^. Since the U bioavailability is governed by its speciation, a change in the speciation of U in the exposure solution as a function of the U concentration could explain that only 0.2% of the total U was incorporated by the cells exposed to 1 µM of U, whereas this percentage increased up to 2% in the case of cells exposed to 125 and 250 µM of U. As isotopic fractionation may occur during any equilibrium reaction between two species, the formation of new bioavailable U species enriched in ^238^U, in the exposure solutions containing 125 and 250 µM of U, could explain the higher δ^238^U (‰) values in cell samples exposed to these U concentrations, with regard to cell samples exposed to 1 and 10 µM of U. Based on the thermodynamic constants available in the literature and from the different literature survey on relevant biological U(VI) species (see ref.^20^ and references therein), the theoretical U speciation diagrams in the exposure solution for U concentrations of 1, 10, 125, and 250 µM were drawn (Fig. 4). Potassium uranyl phosphate phases may be oversaturated and are supposed to precipitate, but the quantitative analysis of U in the exposure solutions through q-ICPMS showed that this precipitation was not significant from 1 to 250 µM of U. This is in agreement with other studies using similar culture media, where no U loss was observed below 300 µM^33^, indicating no precipitation phenomena and a strong interaction of U with the ligands contained in culture medium. As it can be observed, similar speciation diagrams were obtained irrespective of the U concentration. The most significant change was an increase in the percentage of a UO_2_^2+^ complex with one carbonate and human serum albumin (UO_2_HsaCO_3_), ranging from 6.5% at 1 µM of U (Fig. 4a) to 8.5% at 250 µM of U (Fig. 4d) and a similar decrease in the percentage of Ca_2_UO_2_(CO_3_)_3_. Assuming that the increase in the U amount incorporated by the cells was caused by a change in the U speciation, this increase could only be attributed to the displacement of the equilibria towards the formation of UO_2_HsaCO_3_, which would be the bioavailable species. Taking into account that the change in the intracellular δ^238^U (‰) from 10 to 125 µM of U was approximately 0.35‰ (Fig. 2), and that the UO_2_^2+^ transferred to UO_2_HsaCO_3_ from other species corresponded to approximately 25% of the total UO_2_^2+^ in UO_2_HsaCO_3_ (increase from 6.5% at 10 µM of U to 8.5% at 125 and 250 µM of U), the transferred UO_2_^2+^ would need to be approximately 1.4‰ heavier than the UO_2_^2+^ present in UO_2_HsaCO_3_ at 10 µM of U in order to respect the mass balance. Although this possibility cannot be ruled out, such a change in the isotopic signature of the bioavailable U is unlikely, taking into account the small displacement of the UO_2_^2+^ equilibria and the relatively similar coordination environment of UO_2_^2+^ in the different species.

The activation of a U efflux process from a threshold intracellular U concentration is another process that could explain our results. This would imply the intracellular distribution of uranium into different species and the activation of the efflux process triggered by a threshold intracellular U concentration. This is a possibility to be explored, but this would require a much higher isotopic fraction for the efflux than for the uptake process to counterbalance the isotopic fractionation resulting from the U uptake, since the U percentage incorporated by the cells increased by factor 3 from 10 to 125 µM of U in the exposure solution. This seems to be unlikely, since U is the heaviest naturally occurring element, thus typically fractionating less than 1‰ in nature, as found in terrestrial uranium-bearing minerals^39^, even in the case of biologically mediated processes^40^. Following these observations, the most likely hypothesis is that there is a threshold U concentration in the exposure solution triggering a second U uptake pathway, which would become predominant at 125 and 250 µM of U. The first specific transport pathway, responsible for the U uptake at 1 and 10 µM of U, and leading to the intracellular ^235^U enrichment, could also partially account for the U uptake at 125 and 250 µM of U. As suggested in our previous work^20^, this first pathway can result from the interaction between UO_2_^2+^ and a high-affinity transport protein exhibiting higher affinity towards UO_2_^2+^ than the carbonate ligand of the major uranyl species of the exposure solution (Fig. 4). The second uptake pathway would involve a non-specific U uptake pathway leading to a final intracellular U isotopic signature non-significantly different from that of the exposure solution. Indeed, in a previous work dealing with the interaction of a kidney cell line with the radioactive U isotope ^233^U in the 0.2–3.2 µM extracellular concentration range, a multiple transport mechanism was evidenced^41^. The two uptake processes suggested were absorptive endocytosis and the transport mediated by Na-P cotransporters. The knowledge of the equilibria taking place in the U transport through these potential uptake processes would help to rule on the compatibility of these two processes with the intracellular isotopic fractionations determined in our work. Another possibility that might explain the non-significant U isotopic fractionation at 125 and 250 µM is that a steady-state condition was attained once the non-specific U incorporation pathway is activated, leading to similar intra- and extracellular U isotopic signatures. For instance, the input and output of U by diffusion, which is one of the potential non-specific U uptake processes, may explain these results.

In line with the change of the intracellular U isotopic signature, the homeostatic modulation of endogenous elements such as Cu, Fe, and Mn was also dependent on the U exposure concentrations. In particular, a homeostatic variation was evidenced in the case of cells exposed to U concentrations of 125 and 250 µM (Fig. 1). The increase in the intracellular Cu and Fe amounts and the decrease in Mn amounts were induced, while no significant effect was observed for lower U exposure concentrations, except for Fe at 10 µM of U (Fig. 1d). The altered metabolic processes associated with these effects still need to be identified. However, the non-significant change of the Cu and Zn intracellular isotopic signatures as the extracellular U concentration increased, seems to indicate that these processes are not related to the activation of new uptake/efflux pathways of Cu and Zn as a response to the U uptake, though this hypothesis cannot be completely ruled out. As the homeostasis of Cu, Fe, and Mn was disrupted at 125 and 250 µM of U, this alteration might be linked to the activation of the second U uptake pathway. The modification of the activity of the proteins bound to these elements or the modulation of the expression of these proteins, as a response to the intracellular U concentrations, could also explain these results.

## Methods

### Reagents and solutions

All the aqueous solutions were prepared using ultrapure water (resistivity = 18.2 MΩ cm at 25 °C) from a Milli-Q^®^ system (Millipore, Mollsheim, France). Plasma Pure Plus 34–37% HCl and Plasma Pure Plus 67–70% HNO_3_ ultrapure reagents were purchased from SCP Science (Baie–d’Urfé, Canada). IRMM–184 [*n*(^235^U)/*n*(^238^U) = 0.0072623 (22)], IRMM–3636 [*n*(^233^U)/*n*(^236^U) = 1.01906 (16)], IRMM–3702 [*n*(^66^Zn)/*n*(^64^Zn) = 0.56397 (30)] and ERM^®^–AE633 [*n*(^65^Cu)/*n*(^63^Cu) = 0.44563 (42)] isotopic certified reference materials (i–CRM) traceable to SI were purchased from the Institute for Reference Materials and Measurements (IRMM, Geel, Belgium) and were used for the U, Zn, and Cu isotopic analysis by Multi-Collector Inductively Coupled Plasma Mass Spectrometry (MC–ICPMS). A series of 1000 mg L^−1^ standard solutions of Cu, Fe, In, Mg, Mn, P, Sc, U, and Zn (SPEX CertiPrep Group, Longjumeau, France) were used to spike the samples prior to quadrupole Inductively Coupled Plasma Mass Spectrometry (q–ICPMS) analysis.

Eagle’s minimum essential medium (EMEM, ATCC, 30–2003, Manassas, USA), F12 medium (Life Technologies, 21765–029), fetal bovine serum (FBS, ATCC, 30–2020) and penicillin/streptomycin (Gibco-Thermo Fisher Scientific, 15070–063, Darmstadt, Germany) solutions were used to prepare the culture medium for cell growing and exposure experiments. TrypLE Express 1X/EDTA (Gibco-Thermo Fisher Scientific, 12605–010) was used for the trypsinization of cells. Phosphate buffer saline (PBS, pH 7.4) free of CaCl_2_ and MgCl_2_ (Gibco 10010–015) was used to wash the cells after trypsinization. Retinoic Acid (RA) and 12-O-tetradecanoylphorbol-13-acetate (TPA) used for cell differentiation were purchased from Sigma-Aldrich (St. Louis, USA, R2625 and P8139 respectively). A 3 mg mL^−1^ RA solution was prepared in sterile dimethyl sulfoxide (DMSO, Sigma-Aldrich) under a nitrogen atmosphere in opaque tubes and stored at −80 °C. TPA was re-suspended at 3 mg mL^−1^ in sterile DMSO and the solution was stored at −20 °C. NaHCO_3_ analytical reagent (Normapur), anhydrous Na_2_CO_3_, 99.95%, extra pure (Acros Organics-Thermo Fisher Scientific), NaCl puriss. p.a. (Sigma-Aldrich) and tris(hydroxymethyl)aminomethane (TRIS) ultrapure grade ≥99.9% (Sigma-Aldrich) were used to prepare the buffer solution used to prepare the exposure solutions.

### Cell culture and exposure to natural U

Human SH-SY5Y (ATCC, CRL-2266, Batch 59740436) cells were cultured and differentiated into neuron-like cells before U exposure experiments. Cells were grown in 175–cm^2^ flasks at 37 °C in 5% (vol/vol) CO_2_ for 10 days. The culture medium was replaced with fresh medium every 3–4 days. The cells were then passaged by trypsinization using TrypLE Express 1X/EDTA and seeded at 25,000 cells per square centimeter. They were differentiated into neuron-like cells according to a method described elsewhere^42^. For this, the culture medium was replaced with fresh medium containing 10 μM RA, and cells were incubated for 3.5 days. The culture medium was then replaced with fresh medium containing 80 nM of TPA and left for an additional 3.5 days. Differentiated cells were then exposed for 7 days to freshly prepared U exposure solutions with natural U concentrations of 0 (control cells), 1, 10, 125, or 250 µM. Within this period, the solution was replaced once after 3 days. Cells were then trypsinized, collected and washed twice with PBS. Cells pellets were stored at −80 °C until use.

Concerning the exposure solutions, in-house (*Laboratoire de développement Analytique, Nucléaire, Isotopique et Elémentaire* (LANIE), DEN, CEA Saclay) natural uranium oxide powder U_3_O_8_ was dissolved in 0.5 M ultrapure HNO_3_ (SCP Science) to obtain a stock solution at uranium concentration of 151 mM. A buffer solution containing 0.1 mol L^−1^ NaHCO_3_, 0.1 mol L^−1^ Na_2_CO_3_, 0.15 mol L^−1^ NaCl, and 0.05 mol L^−1^ TRIS in ultrapure water was then prepared. An intermediate uranium solution (pH = 8–8.5) was then prepared by a 1:5 dilution of the uranium stock solution into this buffer solution. This dilution was performed by drop-by-drop addition of the uranium stock solution into the buffer solution to avoid U precipitation. Exposure solutions were prepared by diluting the intermediate uranium solution into an appropriate volume of the culture medium, consisting of an equal mix of EMEM and F12 media supplemented with 10% FBS and 1% penicillin/streptomycin. The concentrations of the endogenous elements in the exposure solutions were 19000 ng mL^−1^ of Mg, 45000 ng mL^−1^ of P, 7 ng mL^−1^ of Mn, 420 ng mL^−1^ of Fe, 370 ng mL^−1^ of Zn, and 17 ng mL^−1^ of Cu.

### Multi-elemental quantitative analysis by q-ICPMS

The sample preparation steps and analyses were carried out at the LANIE. Cell samples and exposure solutions were digested in 15 mL polypropylene tubes at room temperature for 48 h with 2 mL of 34–37% HCl and 2 mL of 67–70% HNO_3_. The digested samples were then transferred into Savillex vessels and the solutions were evaporated to dryness at 85 °C using a heating block. A second digestion step in closed Savillex was carried out with 1 mL of 67–70% HNO_3_ at 85 °C for 2 h. The solvent was then evaporated to dryness at 85 °C and the residue was dissolved in 1 mL of 3 M HNO_3_. Then, an aliquot of 0.1 mL of the digested cell samples was diluted with 1.9 mL of 2% HNO_3_ and subjected to the quantitative analysis of U, Mg, P, Mn, Fe, Zn, and Cu by q-ICPMS. The q-ICPMS instrument (X-series, Thermo Fisher Scientific, Darmstadt, Germany) was run under collision cell mode with He gas in the cell at 1.5 mL min^−1^ in order to minimize polyatomic interferences. The isotopes monitored were ^25^Mg, ^31^P, ^55^Mn, ^57^Fe, ^63^Cu, ^64^Zn, and ^238^U. The analysis was performed using the method of standard additions with a single addition of standard^43^. All solutions were spiked with Sc and In at 1.5 ng g^−1^, used as internal standards during q-ICPMS measurements.

### U, Cu, and Zn isotope ratio determinations by MC–ICPMS

The remaining 0.9 mL of the digested cell samples and 1 mL of digested exposure solutions in 3 M HNO_3_ were subjected to a 3-step purification protocol as described elsewhere^26^ to separate U, Cu, and Zn from the matrix components. These solutions were spiked with IRMM-3636 i-CRM before the U purification to correct for any potential U isotopic fractionation during the purification step and for mass bias correction during MC-ICPMS measurements, using the double spike approach^44^. The amount of this i-CRM was adapted to reach signal intensities of 1–2 V for ^233^U^+^ and ^236^U^+^ isotopes. The samples were bracketed with a solution of IRMM-184 i-CRM spiked with IRMM-3636 i-CRM. The purified Cu and Zn fractions were spiked with IRMM-3702 Zn i-CRM and the ERM^®^-AE633 Cu i-CRM, respectively, for mass bias correction using the so-called modified sample-standard bracketing (m-SSB)^45^ approach. The U, Cu and Zn concentrations of the bracketing solutions were systematically matched to those of the samples, being the maximum difference in concentration among solutions lower than 30%.

All isotope ratio measurements were performed with a Neptune Plus MC-ICPMS (Thermo Fisher Scientific) equipped with 9 Faraday detectors and 10^11^ Ω resistor amplifiers. Both conventional and micro-flow sample introduction systems were used. The conventional system consisted of a perfluoroalcoxy micronebulizer operating at around 140 µL min^−1^ (Elemental Scientific, Omaha, USA) coupled to a PC3-SSI Peltier-cooled double-pass spray chamber (Elemental Scientific) at 2 °C. The micro-flow system consisted of an OpalMist nebulizer (Glass Expansion, Melbourne, Australia) working at around 10 µL min^−1^ coupled to an Apex HF desolvation system (Elemental Scientific) and ‘jet’ sampler and X-type skimmer cones adapted to dry plasma conditions^46^. The Apex HF consisted of a cyclonic glass spray chamber heated at 140 °C coupled to a Peltier-cooled spiral condenser at 2 °C. A N_2_ flow was introduced into the Apex HF to improve the sensitivity. The conventional system was used for all the samples, but those containing too small elemental amounts required the more efficient micro-flow system^26^. This was the case of U isotopic analysis in samples containing less than 300 ng of U (i.e., cell samples exposed to 1 µM of natural U) and Cu isotopic analysis in samples containing less than 50 ng of Cu. In some cases, 2 samples were combined, so that the resulting sample contained more than 50 ng of Cu, which allowed performing the analysis with the conventional system. Note that both sample introduction systems provided non-distinguishable results when running standard solutions. Details on the cup configurations used and the corrections applied (mass bias, hydrides, peak tailing, procedural blanks, etc.) are reported in our previous work^26^.

The results are expressed as δ (‰) values, defined as the relative difference in per mil between the isotope ratio of an element in the sample and the same isotope ratio in a reference material. The δ^238^U (‰), δ^65^Cu (‰), and δ^66^Zn (‰) in the samples were determined with regard to the *n*(^238^U)/*n*(^235^U), *n*(^65^Cu)/*n*(^63^Cu), and *n*(^66^Zn)/*n*(^64^Zn) values in the IRMM–184, IRMM–3702, and ERM^®^–AE633 i–CRMs, respectively, as follows^26^:

1 $${\delta }^{238U}({\rm{\textperthousand }})=(\frac{{({R}_{238/235})}_{sample}}{{({R}_{238/235})}_{reference}}-1)\times 1000$$

2 $${\delta }^{65Cu}(\textperthousand )=(\frac{{({R}_{65/63})}_{sample}}{{({R}_{65/63})}_{reference}}-1)\times 1000$$

3 $${\delta }^{66Zn}(\textperthousand )=(\frac{{({R}_{66/64})}_{sample}}{{({R}_{66/64})}_{reference}}-1)\times 1000$$

where ${({R}_{238/235})}_{sample}$, ${({R}_{65/63})}_{sample}$ and ${({R}_{66/64})}_{sample}$ are the *n*(^238^U)/*n*(^235^U), *n*(^65^Cu)/*n*(^63^Cu), and *n*(^66^Zn)/*n*(^64^Zn) isotope ratios determined in the sample, respectively, while ${({R}_{238/235})}_{reference}$, ${({R}_{65/63})}_{reference}$ and ${({R}_{66/64})}_{reference}$ are the *n*(^238^U)/*n*(^235^U), *n*(^65^Cu)/*n*(^63^Cu), and *n*(^66^Zn)/*n*(^64^Zn) isotope ratios in the IRMM–184, IRMM–3702, and ERM^®^–AE633 i–CRMs, respectively.

As explained in our previous work^26^, the expanded uncertainty (${U}_{c}$, k = 2) of individual δ (‰) values was estimated by quadratic propagation of two sources of uncertainty: (i) the within–day measurement reproducibility; and (ii) the reproducibility associated to procedural blank correction.
